# Evaluation of DNA Damage in Common Carp (*Cyprinus carpio* L.) by Comet Assay for Determination of Possible Pollution in Lake Mogan (Ankara)

**DOI:** 10.1100/tsw.2011.140

**Published:** 2011-07-28

**Authors:** İsmet Çok, Onur Kenan Ulutaş, Öncü Okuşluk, Emre Durmaz, Nilsun Demir

**Affiliations:** ^1^ Department of Toxicology, Faculty of Pharmacy, Gazi University, Ankara, Turkey; ^2^ Department of Fisheries and Aquaculture, Faculty of Agriculture, Ankara University, Ankara, Turkey

**Keywords:** Lake Mogan, Cyprinus carpio, common carp, comet assay, genotoxicity

## Abstract

Contamination of the aquatic environment with various concentrations of pollutants results in unexpected threats to humans and wildlife. The consequences of exposure and metabolism of pollutants/xenobiotics, especially carcinogens and mutagens, can be suitably assessed by investigating severe events, such as DNA damage; for example, DNA adducts and DNA strand breaks. One of the commonly used techniques to detect DNA damage in aquatic organisms is single-cell gel electrophoresis (comet assay). This study was carried out using *Cyprinus carpio* in order to identify the possible pollution in Lake Mogan, near Ankara, Turkey, where the city's sewer system and pesticides used in agriculture are believed to be the common causes of pollution. From the comet assay, the tail length (μm), tail intensity (%), and tail moment values of fish caught from Lake Mogan were found to be 31.10 ± 10.39, 7.77 ± 4.51, 1.50 ± 1.48, respectively, whereas for clean reference sites they were found to be 22.80 ± 1.08, 3.47 ± 1.59, 0.40 ± 0.51, respectively. The values are statistically different from each other (*p* < 0.0001, *p* < 0.0001, and *p* < 0.0013, respectively). These results indicate that Lake Mogan may be polluted with substances that have genotoxic effects and constitute an early warning for the lake system. Further detailed research is needed to establish the source of the pollution and the chemicals responsible.

### INTRODUCTION

Aquatic and terrestrial ecosystems are in interaction with the hydrological cycle and with meteorological events. Either through agricultural and industrial activities or through many other human activities, various kinds and amounts of chemicals and xenobiotics pollute the aquatic ecosystem. These pollutants contaminate and accumulate in areas such as small bays, lagoons, lakes, and seas by transportation via various water connections and flows at under/over ground level.

Pollution of the aquatic environment with various chemicals is related to nutritional, reproductive, and behavioral problems that have been occurring in organisms, especially fish, living within the polluted area. As a consequence, this pollution can create significant problems at either the individual or the population level, and lead to a decrease in the population, resulting in unexpected threats to wildlife and to the human consumers of these organisms[1].

By this decade, biomarker studies, used in the evaluation of environmental health as an indicator of toxic effects of environmental pollutants, have become very important and essential. Changes in biochemical level are the "early warning" responses of an organism to environmental alterations and are critically important[2]. One of the most essential features of molecular or cellular biomarkers is that they give a potential forecast about changes, on upper levels, of biological organizations at the population, community, and ecosystem levels. As a consequence, biomarkers can be taken as short-term indications of biological effects that will be seen in the long term[3,4]. There also are some biomarkers that can be used to evaluate the effects that arise from exposure to genotoxic xenobiotics that affect the DNA. DNA damage in aquatic organisms is associated with tumor development, developmental deficiency and retardation, and a decrease in the number of living embryos, larvae, and adults[5,6,7,8]. In recent years, much interest has been shown in measuring DNA single-strand breaks as a sensitive indicator of genotoxicity. The quantity of single-strand breaks is associated with different types of environmental pollutants that have a mutagenic and carcinogenic character[9,10]. Single-cell gel electrophoresis (SCGE), or comet assay, used to measure the number of DNA strand breaks, has found a place in genetic toxicology and in biomonitoring studies as a useful tool in the evaluation of various types of DNA damage and its repair[11,12,13]. The comet technique, used especially in the measurement of DNA damage in mammalian cells, can today be applied to various types of organisms and cells, including fish[14,15,16,17].

Turkey is a Euro-Asian country that is surrounded by seas, while having very rich aquatic environments. However, at the same time, it has many polluted environments. There are studies showing that various lakes in Turkey are polluted with a range of contaminants, especially pesticides[18,19]. Lake Mogan (Fig. 1), located near the Turkish capital of Ankara, is one example of these polluted areas. The lake is nearly 6 $km^2$ in area, with a 2.8-m mean depth. In particular, it is demonstrated in some studies that Lake Mogan has been polluted on a daily basis with residential wastes and agricultural contaminants. It has also been reported that the north side of the lake carries the heaviest pollution load, although pollution was found in all areas along the lake[20,21].

The aim of the present study was to develop the first information concerning the possible biological effects of pollution from clastogenic chemicals in Lake Mogan on organisms living in this lake.

### MATERIALS AND METHODS

#### Chemicals and Media

Frosted microscope slides were obtained from Menzel GmbH (Braunschweig, Germany). Plastics came from Corning Laboratory Sciences (NY, USA), except that microtubes were purchased from Treff Lab (Degersheim, Switzerland). All chemicals for comet assay were purchased from Sigma-Aldrich (Steinheim, Germany), and quality is equal to or above the analytical grade.

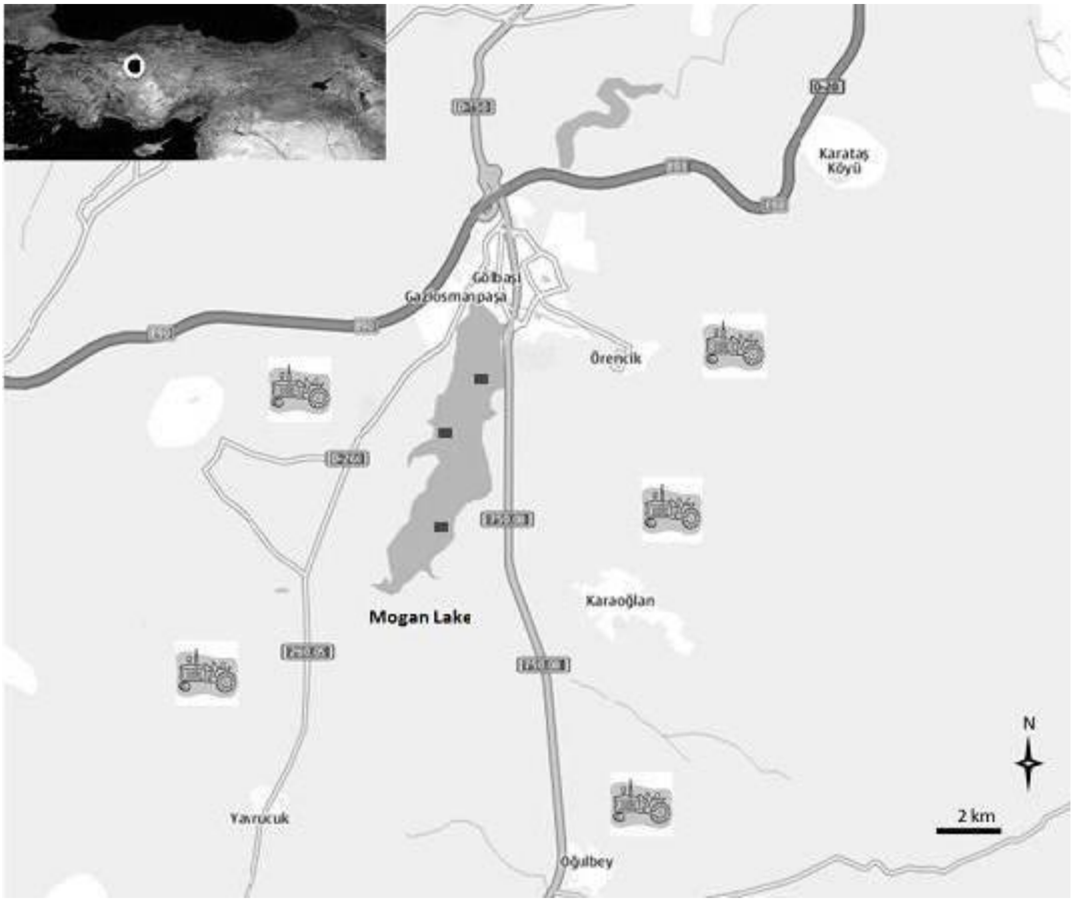

**FIGURE 1.** Map of Turkey showing the location of the study area, Lake Mogan. Squared areas show where sampling was done.

### Animals and Experimental Design

*Cyprinus carpio* L. (common carp), one of the fish species in the lake, is commercially important for the local public and has been used in biomarker studies worldwide[22,23,24]. A total of 30 *C. carpio* were caught by local fishermen from different parts of Lake Mogan randomly and simultaneously (Fig. 1). The blood from the hearts of the fishes near the lake was immediately collected in syringes. The collected blood was then transferred to the laboratory, on ice in a dark box, as quickly as 15 min. The laboratory work started without delay. The comet technique was carried out on the blood of the fish and comparison was made with 15 fish caught from a clean reference site within The Research and Application Fish Farm of Ankara University. The control blood was collected and studied in the same manner.

### Single-Cell Gel Electrophoresis

The alkaline comet assay was performed according to the method of Tice et al.[25], with some modification. About 10 µl of the blood sample collected directly from the heart of common carp was diluted with 500 µl of phosphate-buffered saline. Then 30 µl of the diluted sample was mixed with 100 µl of 0.65% low-melting-point agarose. After that, 100 µl of the mixture was layered on slides precoated with a 0.65% high-melting-point agarose and immediately covered with a coverslip. The slides were kept for 20 min in a refrigerator. After solidification of the gel, coverslips were removed and the slides were immersed in a cold lysing solution (2.5 M NaCl, 100 mM Na2-EDTA, 10 mM Tris, pH 10, with 10% dimethyl sulfoxide and 1% Triton X-100 added fresh) and refrigerated overnight at 4°C. After lysis, the slides were left in the fresh electrophoresis solution (1 mM Na EDTA, 300 mM NaOH, and pH 13.5) for 20 min to allow unwinding. Electrophoresis was performed using the same solution at 300 mA, 25V for 25 min. The slides were than neutralized gently with 0.4 M Tris buffer at pH 7.5 and stained with ethidium bromide.

### Image Analysis of the Slides

After the staining process, a hundred cells were analyzed using double slides, having been selected randomly for examination at 400 (40 × 10) magnification under a fluorescent microscope (Zeiss-Axioskop, Oberkochen, Germany) equipped with an excitation filter of 546 nm and barrier filter of 590 nm, and with a 100-W Hg lamp. The comet assay allows for detection of DNA damage resulting from strand breaks by measuring the migration of DNA fragments from the nucleoid, seemingly resembling a comet. For quantification of DNA damage, the comet assay parameters mean that comet tail length (expressed in µm), tail intensity (% DNA in comet tail), and tail moment were measured and calculated by the Comet Assay III image analysis system (Perceptive Instruments, Steeple Bumpstead, UK). All slides were coded and scored blindly.

### Statistical Analysis

Statistical analyses were performed using the GraphPad InStat 3.05 program for Windows (GraphPad Software, San Diego, CA, USA). Pairwise comparisons for the significance of the differences were performed using the Mann–Whitney U-test. Significance levels of 5% or less were referred to as significant. All *p* values were two-tailed.

## RESULTS AND DISCUSSION

The results of the study are summarized in Table 1. When we compared the comet parameters of the fish from Lake Mogan with those from the reference site, a significant increase in tail length parameter was seen (Lake Mogan, 31.10 ± 10.39 µm; reference site, 22.80 ± 1.08 µm; $p < 0.0001$). The same trend was also noted with the tail moment parameter. The differences between the tail moment parameters of Lake Mogan (1.50 ± 1.48 µm) and the reference site (0.40 ± 0.51 µm) are statistically significant and greater at Lake Mogan ($p = 0.0013$). Tail intensity parameters also show a similar trend. The differences between the tail intensity (%) parameters of Lake Mogan (7.77 ± 4.51 µm) and the reference site (3.47 ± 1.59 µm) are statistically significant, and the DNA damage parameter tail intensity is greater at Lake Mogan ($p < 0.0001$).

**TABLE 1**
**The Results of the Comet Assay**

| | N | Tail Length (µm) | Tail Moment | Tail Intensity (%) |
|---|---|---|---|---|
| Lake Mogan | 30 | 31.10 ± 10.39[a] | 1.50 ± 1.48[b] | 7.77 ± 4.51[a] |
| Reference site | 15 | 22.80 ± 1.08[a] | 0.40 ± 0.51[b] | 3.47 ± 1.59[a] |

[a] $p < 0.0001$, [b] $p = 0.0013$.

The main objective of the study was to try to obtain information about the possible genotoxic effects of pollution on common carp via the comet assay, which has been used widely worldwide as a biomarker tool. Lake Mogan, located 25 km southwest of the capital city of Ankara, is largely surrounded by agricultural areas and has been under the pollution stress of agricultural activities and the city's sewer system. Although local residents and fishermen have become concerned by the increase of pollution in the lake plus the decrease in water level that has occurred, there is no adequate national documentation or research on the determination of cause and of the degree of pollution on the living organisms within this area.

As an environmental biomonitoring tool, the comet assay has been used increasingly by various scientists globally to assess and establish the genotoxic effects of xenobiotics on aquatic organisms[16,26]. The comet technique used here, also known as single-cell gel electrophoresis (the SCGE technique), is a simple, fast, sensitive, and widespread protocol that has been used in recent years to measure and evaluate DNA damage in different cell types even with many organisms[27,28]. One of the most important advantages of the technique is that it can be used with virtually all types of cells, as well as being applicable to fish. Many studies carried out on fish living in sea water as well as in fresh waters can be found in the literature[29,30,31,32,33,34,35,36,37,38].

For the evaluation of exposure to genotoxic agents on fish, studies using methods other than the comet assay (such as the micronucleus test and sister chromatid exchange test) can also be found. In our study, the comet technique was used for its advantages over the micronucleus test and other test methods because (1) only a small number of cells are required, (2) it is faster to conduct, (3) it is more sensitive than other available methods, and (4) it allows an early response evaluation at single cell level on biota[24,30,34]. Some researchers also show that the micronucleus test may demonstrate a negative response to genotoxicants[24]. Balpaeme et al.[33] had positive results for the exposure of brown trout (*Salmo trutta fario*) to PCB 77, both in the micronucleus test and in comet studies; however, with ethyl methane sulfonite (EMS), the micronucleus test showed no induction, while the comet test did.

*Cyprinus carpio* (common carp) is a widely used fish species in the evaluation of the genotoxic potential of agents in *in vitro* studies as well as in environmental studies. To evaluate genotoxic pollutant detection in surface waters, Pellacani et al.[35] determined *in vivo* genotoxicity by the comet assay on common carp, as said assay is cited as a well-established bioindicator organism of water quality. Klobucar et al.[36] dealt with the genotoxicity assessment of fresh water using caged carp (*C. carpio*). In their study, carp from sites with high anthropogenic influence had higher average DNA damage as expressed in both the micronucleus test and comet assay. Of the two, the comet assay appeared to be more sensitive following both caging and aquaria exposure.

Comet assay parameters – tail moment, tail length, and tail intensity – have been used widely by researchers all over the world for determination of DNA damage. As the amount of the damage increases in a cell, more DNA migrates into the tail region and is quantified in terms of increased amount of determined fluorescence in the tail region and tail length. The percentage of DNA in the tail region (tail intensity) has been in use for quantifying DNA strand breakage and is the most advised parameter to use[9]. A major advantage of using tail moment as the index of DNA damage is that both the amount of damaged DNA and the distance of migration of the genetic material in the tail are represented by a single number. Which metric of DNA migration is used somehow depends on the investigator, so in this study, data analysis was performed using tail moment, tail length, and tail intensity, as they can also be easily used to compare with previous and future studies of comet assay.

The main outcome of the comet assay is the detection of excessive DNA strand breaks, a type of lesion of great importance that reflects the recent status of pollution[37], has a temporal nature, and may be removed by the DNA repair system[35]. But chronic exposure to pollutants can lead to accumulation of DNA strand breaks, as the DNA repair capacity of fish cells, an important mechanism to protect DNA integrity, is rather low compared with other species[37,39]. If not repaired, these DNA lesions can initiate a cascade of biological consequences at the cellular, organ, organism, and, ultimately, the community and population levels[29,30].

Estimation of genotoxic effects is essential to any comprehensive study of pollutants in the aquatic environment, and the outcomes of this current study indicate that Lake Mogan may be polluted with xenobiotics that have genotoxic effects, and thus constitute an early warning for the lake system. As there is no research on the determination of pollution in Lake Mogan, a comparison cannot be made in this area. Also, this study is the first pollution evaluation and biomarker study to have been carried out with a biological organism in Lake Mogan.

Although detailed qualitative and quantitative analyses of organic/inorganic chemicals in the waters and sediments of Lake Mogan are not available, the comet assay results point to a high contamination of potential genotoxic pollutants within the lake, and draw attention to the current situation as an early indication of biological effects. Further detailed research is needed to establish the source of the pollution and the chemicals responsible.

### ACKNOWLEDGMENT

This research was supported by the Research Foundation of Gazi University (grant no: 02/2008–18).
